# Airway necrosis and granulation tissue formation caused by *Rhizopus oryzae* leading to severe upper airway obstruction: a case report

**DOI:** 10.3389/fcimb.2024.1366472

**Published:** 2024-03-04

**Authors:** Geng-Jia Chen, Xiao-Bo Chen, Wan-Yuan Rao, Xiao-Yi Pan, Shi-Yue Li, Zhu-Quan Su

**Affiliations:** ^1^ State Key Laboratory of Respiratory Disease, National Clinical Research Center for Respiratory Disease, Guangzhou Institute of Respiratory Health, The First Affiliated Hospital of Guangzhou Medical University, Guangzhou, Guangdong, China; ^2^ Nanshan School of Medical, Guangzhou Medical University, Guangzhou, China

**Keywords:** *Rhizopus oryzae*, tracheal stenosis, mucosal necrosis, amphotericin B, bronchoscopy

## Abstract

Pulmonary Mucormycosis is a fatal infectious disease with high mortality rate. The occurrence of Mucormycosis is commonly related to the fungal virulence and the host’s immunological defenses against pathogens. Mucormycosis infection and granulation tissue formation occurred in the upper airway was rarely reported. This patient was a 60-year-old male with diabetes mellitus, who was admitted to hospital due to progressive cough, sputum and dyspnea. High-resolution computed tomography (HRCT) and bronchoscopy revealed extensive tracheal mucosal necrosis, granulation tissue proliferation, and severe airway stenosis. The mucosal necrotic tissue was induced by the infection of *Rhizopus Oryzae*, confirmed by metagenomic next-generation sequencing (mNGS) in tissue biopsy. This patient was treated with the placement of a covered stent and local instillation of amphotericin B via bronchoscope. The tracheal mucosal necrosis was markedly alleviated, the symptoms of cough, shortness of breath, as well as exercise tolerance were significantly improved. The placement of airway stent and transbronchial microtube drip of amphotericin B could conduce to rapidly relieve the severe airway obstruction due to Mucormycosis infection.

## 1 Introduction

Pulmonary Mucormycosis is a fatal infectious disease with high mortality rate. The occurrence of Mucormycosis is commonly related to the fungal virulence and the host’s immunological defenses against pathogens. Mucormycosis is clinically classified into different types based upon the site of infection, including pulmonary Mucormycosis, rhino-orbital-cerebral Mucormycosis, cutaneous Mucormycosis, gastrointestinal Mucormycosis and disseminated Mucormycosis. Here we reported a case of severe upper airway stenosis caused by Mucormycosis infection, presenting tracheal mucosal necrosis and granulation tissue proliferation.

## 2 Case presentation

**Figure 1:**
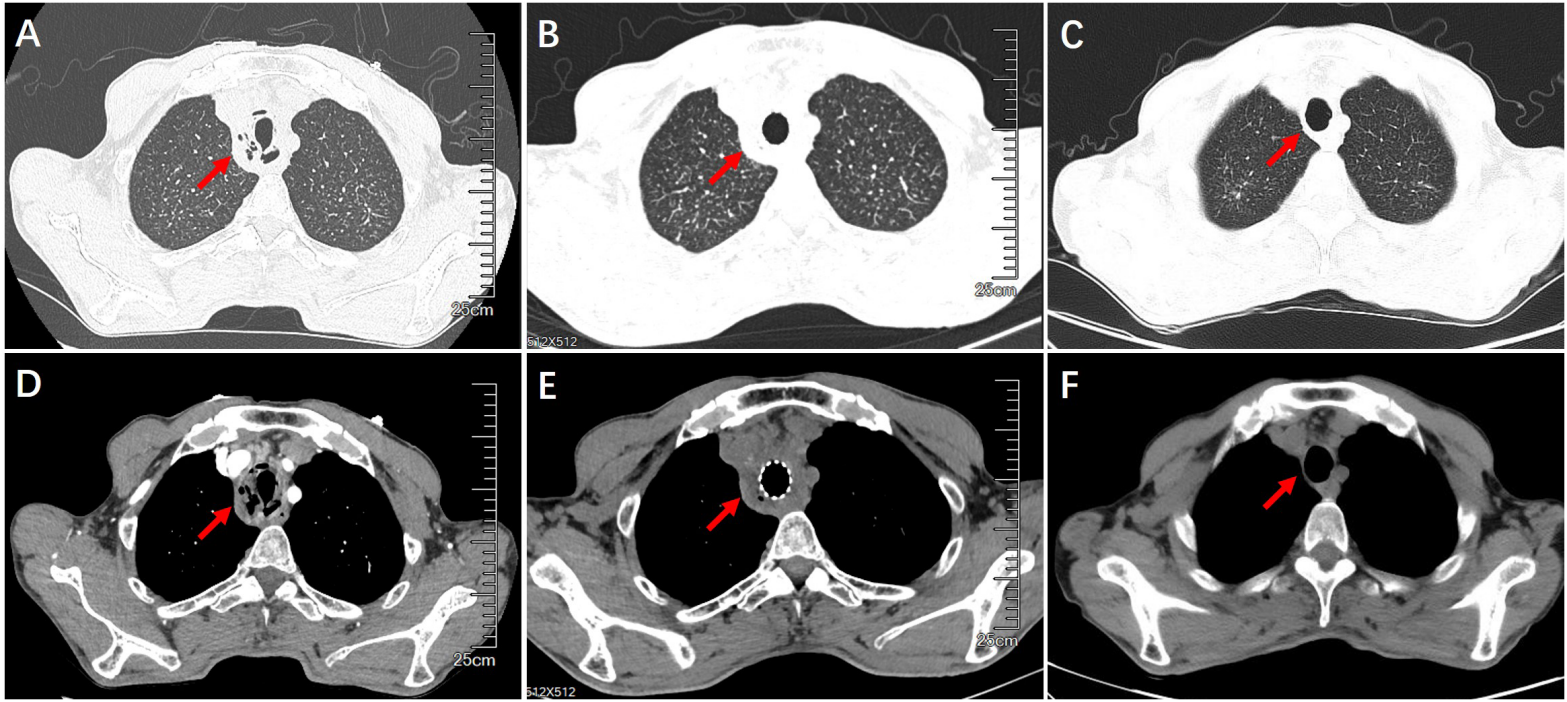
Chest CT imaging on airway structural changes before and after treatment. **(A, D)** Airway wall thickening, necrosis and luminal obstruction before treatment. **(B, E)** Airway patency after the placement of airway stent. **(C, F)** Improvement of tracheal wall thickening and luminal narrowing after interventional treatment with airway stent withdraw.

**Figure 2:**
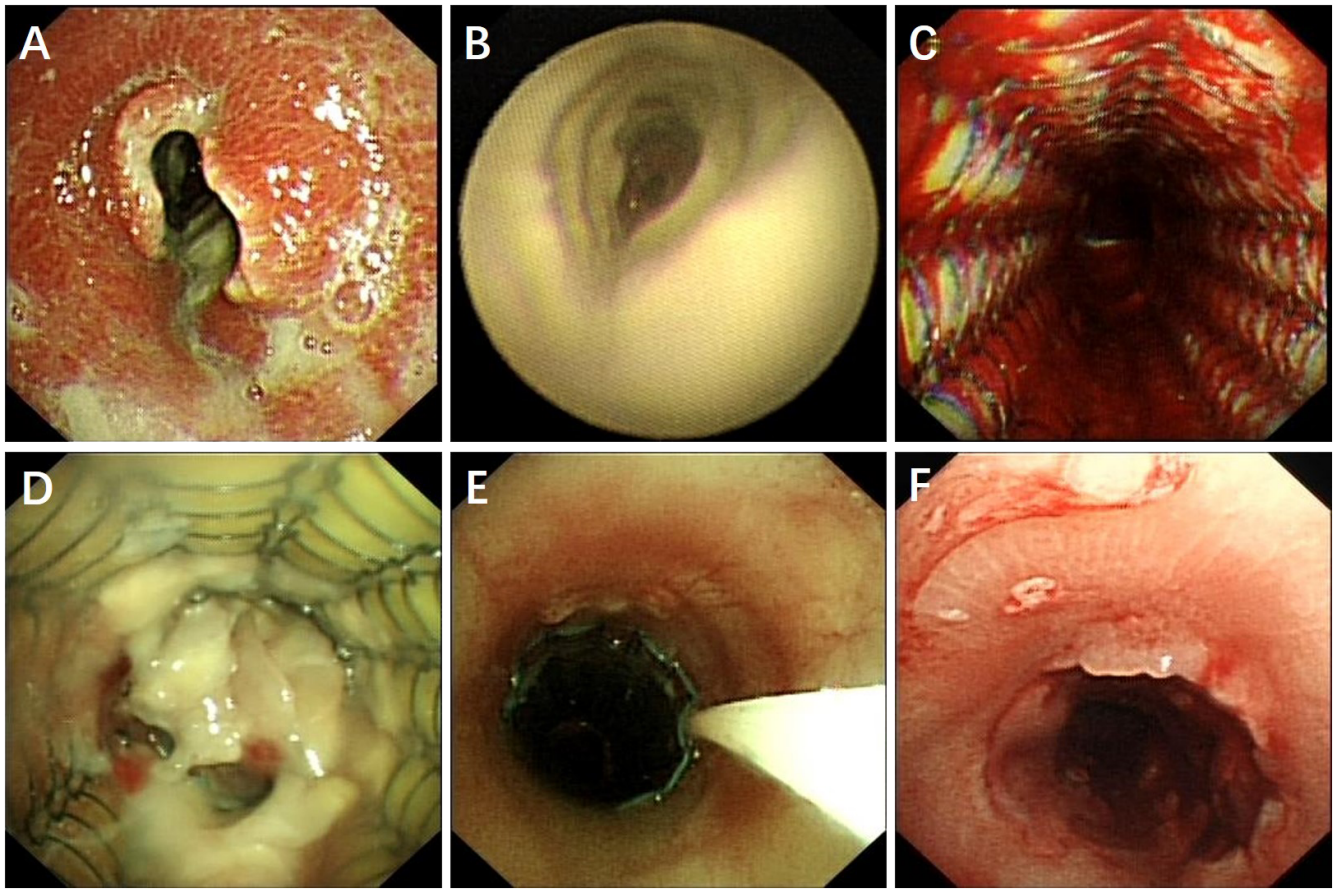
Bronchoscopic finding of tracheal *Rhizopus Oryzae* infection before and after treatment. **(A, B)** Tracheal mucosal necrosis, granulation tissue proliferation and severe airway necrosis before treatment. **(C)** Airway stent placement to maintain luminal patency. **(D)** Patient did not respond to intravenous amphotericin B treatment, airway necrosis and airway obstruction recurred. **(E)** Topically dripping amphotericin B between the stent and tracheal wall by microtubule via bronchoscope. **(F)** Patent airway with a small amount of granulation tissue and an intact tracheal mucosa repair without necrotic adherence.

A 60-year-old male, who worked as a miner with long-term dust exposure, had a history of type 2 diabetes mellitus. Four months ago, this patient developed symptoms of cough with a large amount of sputum, accompanied by intermittent fever and progressive shortness of breath. The laboratory tests demonstrated increased blood glucose (17.02 mmol/L), glycosylated hemoglobin (HbA1c) 17.5%, procalcitonin (0.58 ng/mL), and white blood cell counts (11.30×109/L) with 79.3% neutrophils when he was admitted to hospital. Liver and renal function, Antineutrophil Cytoplasmic Antibodies (ANCA), as well as molecular assay for tuberculosis were otherwise unremarkable. The chest CT and bronchoscopy suggested extensive mucosal necrosis with granulation tissue formation and significant luminal narrowing in the middle and upper trachea (**Figures 1A, D**, **2A, B**). Considering the mucosal necrosis and granulation tissue formation had induced to severe tracheal stenosis, a covered metallic stent was urgently implanted to relieve airway obstruction (**Figures 1B, E**, **2C**). Afterwards, the mNGS testing was performed in the airway mucosal necrotic tissue, suggesting tracheal *Rhizopus oryzae* infection.

Based on the diagnosis of airway *Rhizopus oryzae* infection, a full dose and course of amphotericin B intravenous drip combined with nebulized inhalation treatment was given. Whereas, the one-month CT reexamination demonstrated no change in tracheal wall thickening and luminal stenosis, whilst, the bronchoscopy unexpectedly revealed the progressive development of mucosal necrosis and severe airway obstruction (**Figure 2D**). In an attempt to enhance the drug penetration to the tracheal lesion, amphotericin B (5mg, mixed with saline 20ml) was topically dripped between the stent and tracheal wall by microtubule via bronchoscope once a week (**Figure 2E**), which might be conducive to prolonging the contact time of the anti-fungal administration. Whilst, oral posaconazole was administered as sequential therapy for the treatment of *Rhizopus Oryzae* infection. Chest CT and bronchoscopy follow-up were performed after 1 month, showing a gradual reduction of mucosal necrotic tissue in the airway, as well as an improvement of the tracheal wall thickening (**Figures 1C, F**, **2C**). After 3 months of treatment, the tracheal metallic stent was removed, the bronchoscopy revealed a patent airway with a small amount of granulation tissue and an intact tracheal mucosa repair without necrotic adherence (**Figures 1C, F**, **2F**). Accordingly, the patient’s symptoms of sputum and shortness of breath were relieved, while the quality of life and exercise tolerance were significantly improved.

## 3 Discussion

Pulmonary Mucormycosis is a fatal fungal infection with a rapidly progressive course and 49.8% -72.1% mortality rate (Cornely et al., 2019; Muthu et al., 2021), most cases present acute onset without specific clinical presentation (Danion et al., 2015). Mucormycosis is commonly caused by ubiquitous environmental moulds with a global distribution, including *Rhizopus oryzae*, *Mucor racemosus*, *Mucor corymbifer*, *Absidia ramosa* and *Rhizopus arrhizus* (Stone et al., 2021). Mucormycosis mainly occurs in immunocompromised hosts, the common risk factors include diabetes mellitus, malignancy with neutropenia, solid organ transplantation, immunosuppression, HIV infection, trauma/surgery, and malnutrition (Steinbrink and Miceli, 2021). In this case, the patient had poorly controlled diabetes, which was regarded as immunocompromised population and vulnerable to Mucormycosis infection.

The diagnosis of Mucormycosis infection is commonly based on the pathogen detection and histopathological findings. However, the sputum and bronchoalveolar lavage fluid culture was reportedly long time consuming and less than 5% of culture positive rate, making it a potential risk of disease progression and high mortality. Metagenomics next-generation sequencing (mNGS) detection (Han et al., 2019; Zheng et al., 2021; Diao et al., 2022), considered to be more sensitive than routine bacterial culture (Zhang et al., 2020), could be of great value in the diagnosis of acute and critical infections or mixed infections (Brown et al., 2018). In this case, since the patient suffered from progressive and severe airway stenosis caused by mucosal necrosis and granulation tissue formation, the early diagnosis and treatment was urgently needed and served as the key to the improvement of clinical prognosis. Whereafter, the mNGS in mucosal necrotic tissue was performed to identify *Rhizopus oryzae* infection, which might be responsible for the invasive damage to the airway mucosal structure. Mucormycosis is characterized by perivascular erosion, the mycelium could invade the microvessel to form thrombi, causing tissue ischemia and hypoxia and eventually, hemorrhage and necrosis.

Nasal-orbital-brain and lung are the most common site of Mucormycosis infection (Skiada et al., 2020), which occur in patients with diabetes and malignancy, as well as those with solid organ transplantation and glucocorticoid use, respectively. Whereas, in this case, Mucormycosis infection and granulomas formation occurred in the upper airway, which was rarely reported in the previous literature. It should be highlighted that the formation of pseudomembrane due to tracheal mucosal necrosis, and granulation tissue proliferation could lead to progressive airway obstruction and life-threatening dyspnea. Noteworthily, Mucormycosis infection related airway stenosis should be distinguished from tracheobronchial tuberculosis, invasive aspergillosis, and vasculitis.

Amphotericin B is regarded as the first-line treatment for pulmonary Mucormycosis infection. In this case, the patient with mucosal necrosis and pseudomembrane formation did not respond to the standardized intravenous and atomized amphotericin, which might due to the inadequate penetration of the drug into the lesion. Drug concentration of Amphotericin B in bronchial secretions is much less than that of contemporaneous blood concentrations, making it a natural choice for clinicians to explore the topical application of Amphotericin B. Amphotericin B could be applied through the airway lumen, mainly consisting of transoral nebulized inhalation (Kuiper and Ruijgrok, 2009; Alissa et al., 2021) and transbronchoscopic local instillation. In this case, transbronchoscopic microtube drip of amphotericin B was used outside the metallic stent (gap between the stent and the airway wall), in an attempt to increasing the drug concentration in the airway wall, and reducing the loss of the drug in the airway. Finally, the patient responded well to the transbronchial topical drip of amphotericin B treatment, the airway mucosal pseudomembrane was gradually eliminated, while the airway narrowing was significantly relieved. In sum, transtracheobronchial microtube drip of amphotericin B might relatively reduce the adverse drug reaction, furthermore, the drip administration between the outer side of the stent and the airway wall could conduce to prolong the duration of drug action.

## 4 Conclusion

We report a case of severe upper airway stenosis due to Mucormycosis infection, the placement of airway stent and transbronchial microtube drip of amphotericin B could conduce to rapidly relieve the airway obstruction, as well as prolong the duration of drug action to improve the antifungal therapeutic effect.

## Data availability statement

The original contributions presented in the study are included in the article/supplementary material. Further inquiries can be directed to the corresponding author.

## Ethics statement

The studies involving humans were approved by the First Affiliated Hospital of Guangzhou Medical University Medical Ethics Committee. The studies were conducted in accordance with the local legislation and institutional requirements. The participants provided their written informed consent to participate in this study. Written informed consent was obtained from the individual(s) for the publication of any potentially identifiable images or data included in this article.

## Author contributions

G-JC: Conceptualization, Data curation, Formal analysis, Writing – original draft, Writing – review & editing. X-BC: Conceptualization, Data curation, Formal analysis, Writing – original draft, Writing – review & editing. W-YR: Conceptualization, Data curation, Formal analysis, Writing – original draft, Writing – review & editing. X-YP: Conceptualization, Data curation, Formal analysis, Writing – original draft, Writing – review & editing. S-YL: Supervision, Validation, Writing – original draft, Writing – review & editing. Z-QS: Funding acquisition, Project administration, Resources, Supervision, Validation, Writing – original draft, Writing – review & editing.
